# Oxidative phenylamination of 5-substituted 1-hydroxynaphthalenes to *N*-phenyl-1,4-naphthoquinone monoimines by air and light “on water”

**DOI:** 10.3762/bjoc.10.255

**Published:** 2014-10-22

**Authors:** Julio Benites, Juan Meléndez, Cynthia Estela, David Ríos, Luis Espinoza, Iván Brito, Jaime A Valderrama

**Affiliations:** 1 Facultad de Ciencias de la Salud, Universidad Arturo Prat, Casilla 121, Iquique, Chile; 2 Instituto de Ciencias Exactas y Naturales (ICEN), Universidad Arturo Prat, Casilla 121, Iquique, Chile; 3 Facultad de Química, Universidad Técnica Federico Santa María, Casilla 110-V, Valparaíso, Chile; 4 Departamento de Química, Facultad de Ciencias Básicas Universidad de Antofagasta, Casilla 170, Antofagasta, Chile

**Keywords:** 1,4-naphthoquinone monoimines, on water, oxidative coupling, rose bengal, solar radiation

## Abstract

A number of *N*-phenyl-1,4-naphthoquinone monoimines **6**–**10** were prepared by on-water oxidative phenylamination of 1,5-dihydroxynaphthalene (**1**) and 5-acetylamino-1-hydroxynaphthalene (**5**) with oxygen-substituted phenylamines under aerobic conditions and either solar or green LED radiation, in the presence of rose bengal as singlet oxygen sensitizer. As compared to the conventional oxidative phenylamination procedures, this novel synthetic method offers the advantage of aerobic conditions “on water” instead of hazardous oxidant reagents currently employed in aqueous alcoholic media.

## Introduction

**Figure 1:**
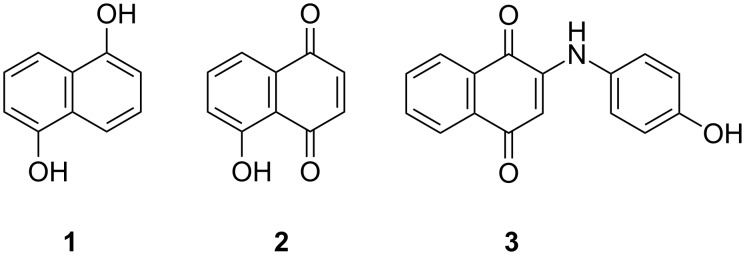
Structures of compounds **1**–**3**.

The growing demand for the application of environmentally friendly technologies has led, in the last decades, to a great deal of research effort to develop low-impact alternative synthetic methods as well as to replace toxic and harmful solvents by more environmentally benign ones [1–6]. In this context, the sunlight-induced synthesis of 5-hydroxy-1,4-naphthoquinone (**2**, juglone, Figure 1) by sensitized phothooxygenation of 1,5-dihydroxynaphthalene (**1**, 1,5-DHN) [7] and the preparation of 2-phenylamino-1,4-naphthoquinones by reaction of 1,4-naphthoquinones with phenylamines “on water” [8] represent two valuable examples on the scope of green methodologies in the field of quinone synthesis.

1,4-Naphthoquinones possessing a substituted amino group in the 2-position have been the subject of study for many years due to their use in a variety of medical and biological applications. A well-documented procedure to prepare 2-amino-1,4-naphthoquinone derivatives is based on the reaction of amines with 1,4-naphthoquinone [9]. This atom-economical method to construct aminoquinones occurs through an addition–oxidation reaction sequence [10]. In a previous work we described that 2-(4-hydroxyphenyl)amino-1,4-naphthoquinone (**3**), prepared by acid-induced amination of 1,4-naphthoquinone with 4-hydroxyphenylamine in ethanol, strongly inhibit the proliferation of cancer cells [11–12]. These stimulating results were obtained by using prostate (DU-145), bladder (T24) and breast (MCF-7) cancer cells. These results and our continuous interest in the synthesis of aminoquinones for antiproliferative evaluation [11–13], prompt us to explore a straightforward access to potential antiproliferative aminojuglone-analogues of compound **3**. The study led us to discover a potential and green route for the preparation of *N*-phenyl-1,4-naphthoquinone monoimines by on-water oxidative coupling reaction of 1-hydroxynaphthalene derivatives with oxygen-substituted phenylamines under solar light or green LEDs radiation, rose bengal as singlet oxygen sensitizer and aerobic conditions.

## Results and Discussion

**Scheme 1:**
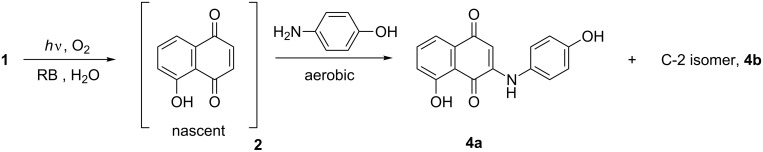
Hypothetical one-pot synthesis of compound **4a** and/or **4b**.

Our goal was to develop a simple method for the preparation of phenylaminojuglone analogues of compound **3** via a green one-pot procedure “on water”, based on the hypothetical sequence reactions outlined in Scheme 1. According to this sequence the nascent juglone (**2**), generated by sensitized solar-induced photooxygenation of 1,5-DHN (**1**), might undergo in situ phenylamination followed by aerobic oxidation to give the expected target aminojuglone **4a** and/or **4b**. In order to evaluate the reactions involved in this hypothetical strategy, the solar photosensitized photooxygenation of 1,5-DHN (**1**) and the amination of juglone (**2**) with 4-hydroxyphenylamine on water were examined separately. It is noteworthy that similar reactions have been recently reported by using a one-flask procedure where juglone **2** was first produced by photoinduced photooxygenation of 1,5-DHN (**1**) in CH_2_Cl_2_/MeOH and then in the same flask, the arylamination of **2** to the respective aminoquinones was accomplished [14].

The on-water photooxygenation of 1,5-DHN (**1**) was carried out for 5 h in round-bottom flasks, under the presence of 1.6 mmol % of rose bengal (RB) as singlet oxygen sensitizer, by using sunlight (Canchones Experimental Center in Iquique/Chile, latitude 20°26´43.80´´ S, 990 m above sea level) and green LEDs as photochemical sources. Meanwhile, a gently stream of air was bubbled through the solutions. Under these conditions juglone (**2**) was isolated in 81% (sunlight) and 55% (LEDs) yields. On the other hand, the reaction of juglone (**2**) with 4-hydroxyphenylamine, performed in water suspension at room temperature, gives rise compounds **4a** and **4b** in 45% and 16% yields, respectively. The structures of compounds **4a** and **4b** were fully established on the basis of their nuclear magnetic resonance (^1^H NMR, ^13^C NMR, 2D NMR) and high resolution mass spectrometry (HRMS). The position of the nitrogen substituent in these aminoquinones was determined by means of HMBC experiments.

Encouraged by these results, the preparation of aminojuglones **4a** and **4b** was attempted by employing the hypothetical one pot procedure outlined in Scheme 1. A suspension of equimolar amounts of 1,5-DHN (**1**) and 4-hydroxyphenylamine in water was irradiated by sunlight for 5 h, in the presence of RB. Work-up of the reaction mixture afforded a dark brown solid compound, which was fully characterized as 5-hydroxy-4-((4-hydroxyphenyl)imino)naphthalene-1(4*H*)-one (**6**, 98% yield). Under similar conditions, but using green LEDs light radiation, compound **6** was isolated in 80% yield.

**Figure 2:**
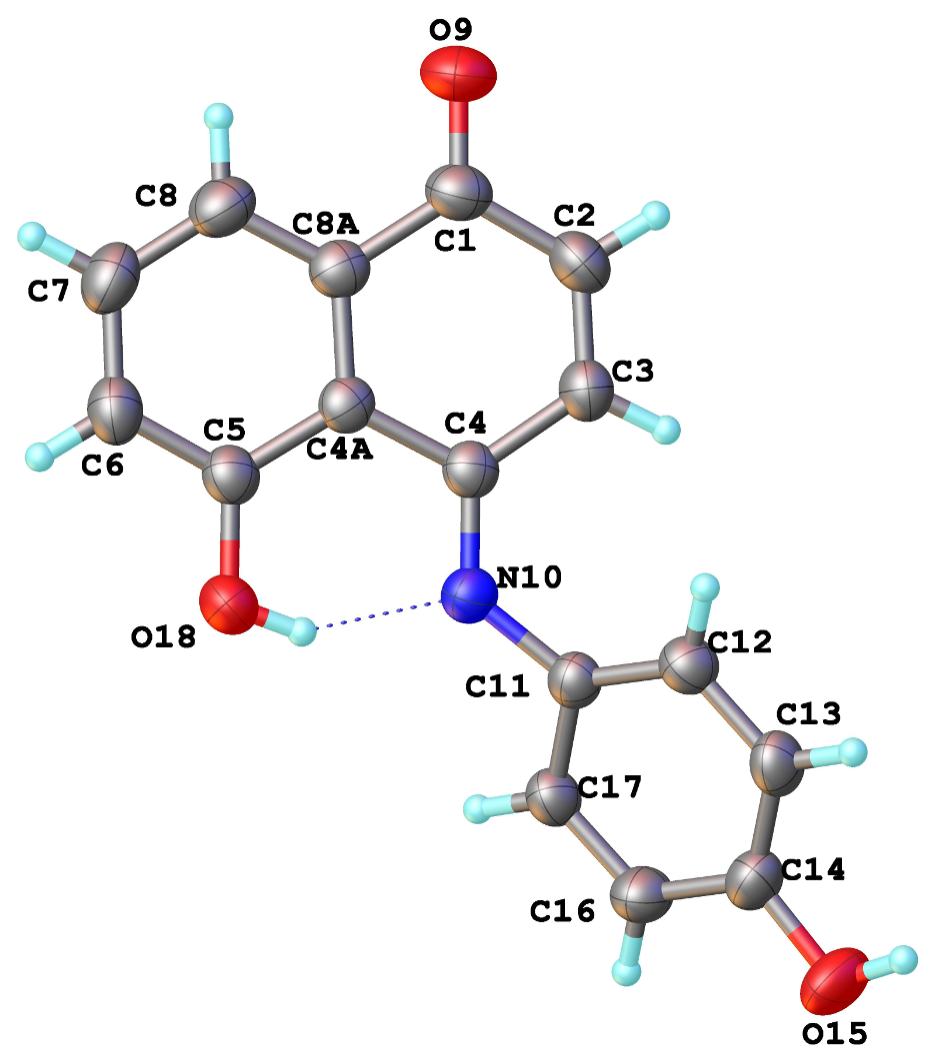
Structure of compound **6** determined by single crystal X-ray diffractometry.

The structure of **6** was established by means of 1D and 2D NMR and by X-ray crystallographic studies (Figure 2 and Table S1 in Supporting Information File 1). This unexpected but interesting result indicates that on-water oxidative amination reaction of 1,5-DHN (**1**) with 4-hydroxyphenylamine to produce compound **6** is more favorable than the photooxygenation reaction to give juglone (**2**).

The *N*-phenylquinone imines are considered as an important class of compounds because of their properties as dyes for optical devices [15], antioxidants [16], anticancer agents [17] and precursors of liquid crystalline materials [18]. With regard to the synthesis of *N*-phenyl-1,4-naphthoquinone monoimines, Bukhtoyarova et al. [19], has described their preparation by reaction of 1,5-DHN (**1**) with phenylamines using oxidants such as K_3_Fe(CN)_6_, HIO_3_ and NaIO_4_ in aqueous alcohol. According to these authors, the oxidative phenylamination mechanism involves a radical cation intermediate generated by an electron transfer process from the phenylamine to the oxidant. Further electrophilic substitution at the 4-position of compound **1** followed by oxidation of the aminonaphthol intermediate, yield the *N*-phenyl-1,4-naphthoquinone-4-imines. A mechanism involving radical cation intermediates is supported by the rather high product yields resulting from the reaction of 1,5-DHN (**1**) with electron-donor substituted phenylamines.

**Scheme 2:**
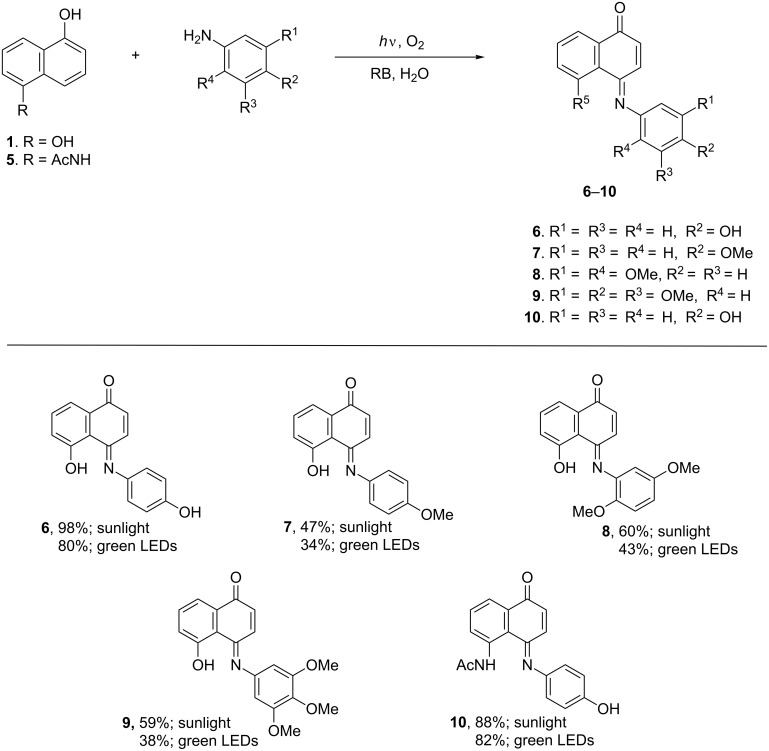
Evaluation of the substrate scope using RB as oxygen (^1^O_2_) sensitizer “on water”.

Given the totally green and high yield access to compound **6** from 1,5-DHN (**1**) and 4-hydroxyphenylamine, we decided to study the reaction scope to include 5-acetylamino-1-hydroxynaphthalene (**5**) as a 1-hydroxynaphthalene substrate and phenylamines containing electron-donor and acceptor substituents such as: 4-methoxyphenylamine, 2,5-dimethoxyphenylamine, 3,4,5-trimethoxyphenylamine and 4-nitrophenylamine. The results obtained under standard conditions using sunlight and green LEDs are outlined in Scheme 2. Since no reaction was observed in the assay with 4-nitrophenylamine it may be concluded that the oxidative arylamination of 1,5-DHN (**1**) is favorable with arylamines containing electron-donor substituents.

**Scheme 3:**
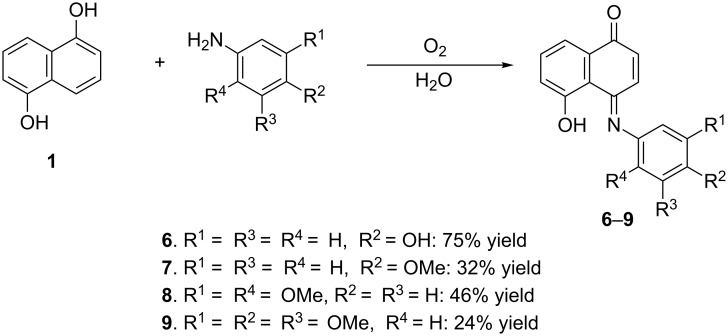
Evaluation of the oxidative coupling in the absence of RB, on water.

Bearing in mind that the formation of the quinone monoimines **6**–**10** under the above experimental conditions requires aerial oxidation reactions, most likely mediated by oxygen species such as singlet oxygen [20], we examined the effect of histidine, a well-known singlet oxygen scavenger [21], on the formation of iminoquinone **6** from 1,5-DHN (**1**) and 4-hydroxyphenylamine. When the experiments were performed under standard conditions by using sunlight or green LEDs in the presence of histidine, compound **6** was obtained in 69 and 62% yield, respectively. By comparing these results to that observed in the absence of histidine, it may be suggested that singlet oxygen is involved in the oxidative coupling reaction to produce compound **6**. In addition, experiments on the oxidative coupling reaction of 1,5-DHN (**1**) with oxygen-substituted phenylamines were performed in the absence of RB under in-door daylight illumination. The results are outlined in Scheme 3 and showed lower yields as compared to that obtained by using RB. Taking into account all the experiments we have done, it may be concluded that singlet oxygen is involved in the oxidative amination reaction, albeit with partial contribution to the aerial oxidation processes.

## Conclusion

In summary, we have developed a green procedure for the synthesis of *N*-phenyl-1,4-naphthoquinone monoimines, in moderate to excellent yields, from 5-substituted 1-hydroxynaphthalenes and oxygen-substituted phenylamines using air, sunlight or green LEDs, rose bengal as sensitizer “on water”. This method offers operational simplicity, mild conditions and environmental friendliness as the features of this protocol. Further investigations to expand the scope and potential of this methodology are under way in our laboratory.

## Supporting Information

File 1 Experimental procedures, characterization data, copies of the NMR spectra of compounds **4a**, **9**, **10** and X-ray view of compound **6**.
