# Supplementary material for: Oxidative phenylamination of 5-substituted 1-hydroxynaphthalenes to N-phenyl-1,4-naphthoquinone monoimines by air and light “on water”
Source: Beilstein J Org Chem. 2014 Oct 22;10:2448–52. doi: 10.3762/bjoc.10.255 (PMC4222432; doi:10.3762/bjoc.10.255)
Supplement: File 1 — Experimental procedures, characterization data, copies of the NMR spectra of compounds 4a, 9, 10 and X-ray view of compound 6. [file Beilstein_J_Org_Chem-10-2448-s001.pdf]

Supporting Information

for

Oxidative phenylamination of 5-substituted 1-hydroxynaphthalenes to *N*-phenyl-1,4-naphthoquinone monoimines by air and light “on water”

Julio Benites*[1,2], Juan Meléndez[1], Cynthia Estela[1], David Ríos[1], Luis Espinoza[3], Iván Brito[4] and Jaime A. Valderrama[1,2]

Address: [1]Facultad de Ciencias de la Salud, Universidad Arturo Prat, Casilla 121, Iquique, Chile, [2]Instituto de Ciencias Exactas y Naturales (ICEN), Universidad Arturo Prat, Casilla 121, Iquique, Chile, [3]Facultad de Química, Universidad Técnica Federico Santa María, Casilla 110-V, Valparaíso, Chile and [4]Departamento de Química, Facultad de Ciencias Básicas Universidad de Antofagasta, Casilla 170, Antofagasta, Chile

Email: Julio Benites* - julio.benites@unap.cl

* Corresponding author

Experimental procedures, characterization data, copies of the NMR spectra of compounds 4a, 9, 10 and X-ray view of compound 6.

Page Contents

2 Experimental

2 Compounds **4a** and **4b**

5 X-ray crystallography of compound **6**

7 ^{1}H NMR and ^{13}C NMR spectra of compounds **4a** (HMBC), **9** and **10**

Experimental:

General

All reagents were commercially available reagent grade and were used without further purification. Melting points were determined on a Stuart Scientific SMP3 apparatus and are uncorrected. ^{1}H-NMR spectra were recorded on Bruker AM-400 instrument. ^{13}C-NMR spectra were obtained at 100 MHz. Bidimensional NMR techniques and DEPT were used for signal assignment. Chemical shifts are expressed in ppm downfield relative to tetramethylsilane and the coupling constants (*J*) are reported in Hertz. HRMS data for all final compounds were obtained using a LTQ-Orbitrap mass spectrometer (Thermo-Fisher Scientific, MA 02454, USA) with the analysis performed using an APCI source operated in positive mode. Silica gel Merck 60 (70–230 mesh) was used for preparative column chromatography and TLC aluminum foil $60F_{254}$ for analytical TLC.

Procedure for synthesis of compound 4a and 4b

A suspension of 1,5-dihydroxynaphthalene (**1;** 1.25 mmol), rose bengal (20 mg; 0.02 mmol) as sensitizer and water (150 mL), in a round bottom flask, was exposed to direct sunlight for 5 h while a gentle stream of air is bubbled through the solution. Then is added 4-hydroxyphenylamine (1.5 mmol), and the solution was stirred at rt for 4 h. Work up followed by column chromatography over silica gel (2.5:1.5 petroleum ether/ethyl acetate) yield pure compounds **4a** and **4b**.

Spectroscopic data

5-Hydroxy-3-((4-hydroxyphenyl)amino)naphthalene-1,4-dione (***4a*)**: Isolated yield 45%; brown solid, mp 247-249 °C; IR (KBr) $\nu_{máx}$ cm^{-1}: 3384 (O-H), 3340 (O-H), 3286 (N-H), 1630 (C=O), 1619 (C=O). ^{1}H-NMR (400 MHz, DMSO-d_6): δ 5.82 (s, 1H, 2-H), 6.85 (d, 2H, *J* = 8.5 Hz, 3'-H + 5'-H), 7.16 (d, 2H, *J* = 8.5 Hz, 2'-H + 6'-H), 7.23 (d, 1H, *J* = 8.4 Hz, 6-H), 7.45 (d, 1H, *J* = 7.3 Hz, 8-H), 7.72 (dd, 2H, *J* = 7.8, 7.9 Hz, 7-H), 8.27 (s, 1H, 4'-OH), 9.0 (s, 1H, NH), 9.54 (s, 1H, 5-OH); ^{13}C-NMR (100 MHz, DMSO-d_6): δ 101.54, 114.67, 116.26 (2C), 117.94, 122.36, 126.25 (2C), 129.25, 133.60, 137.91, 147.45, 155.84, 160.90,

181.80,186.18. HRMS (APCI) calcd. for $C_{16}H_{11}NO_4$: 281.06881 $[M+H]^+$; found 281.07541.

5-Hydroxy-2-((4-hydroxyphenyl)amino)naphthalene-1,4-dione (***4b***)**:** Isolated yield 16%; brown solid, mp 241-243 °C; IR (KBr) $\nu_{máx}$ cm^{-1}: 3383 (O-H), 3339 (O-H), 3286 (N-H), 1631 (C=O), 1619 (C=O). ^{1}H-NMR (400 MHz, DMSO-d_6): δ 5.82 (s, 1H, 3-H), 6.85 (d, 2H, J = 8.6 Hz, 3'-H + 5'-H), 7.16 (d, 2H, J = 8.6 Hz, 2'-H + 6'-H), 7.22 (d, 1H, J = 8.3 Hz, 6-H), 7.45 (d, 1H, J = 7.3 Hz, 8-H), 7.72 (dd, 2H, J = 7.7, 8.1 Hz, 7-H), 9.0 (s, 1H, NH), 9.56 (s, 1H, 4'-OH), 11.45 (s, 1H, 5-OH); ^{13}C-NMR (100 MHz, DMSO-d_6): δ 101.56, 114.67, 116.27 (2C), 117.93, 122.36, 126.24 (2C), 129.26, 133.61, 137.89, 147.44, 155.85, 160.92, 181.80, 186.16; HRMS (APCI) calcd. for $C_{16}H_{11}NO_4$: 281.06881 $[M+H]^+$; found 281.07527.

General procedure for the synthesis of compounds 6-10

A suspension of 1,5-dihydroxynaphthalene (**1**) or 5-acetylamino-1-hydroxynaphthalene (**5**; 1.25 mmol), rose bengal (20 mg), the phenylamine required (1.25 mmol) and water (150 mL), into a round bottom flask, was vigorously stirred at room temperature. Then, the solution was irradiated by solar light or Light Emitting Diode lamps (LED: InGaN, 0.768 W, 42.24 lm, 530 nm) for 5 h at the same time a gently stream of air is bubbled through the solution. The reaction mixture was extracted with ethyl acetate (2 x 20 mL), the dry extract was evaporated under vacuum and the residue was chromatographed on silica gel (3:1 petroleum ether/ethyl acetate) to give pure the respective *N*-phenyl quinone imines.

Spectroscopic data

5-Hydroxy-4-((4-hydroxyphenyl)imino)naphthalen-1(4H)-one (**6**): Prepared from **1** and 4-hydroxyphenylamine, in 98 and 80% yield by using solar light and green LEDs respectively; brown solid, mp 149-151 °C; IR (KBr) $\nu_{máx}$ cm^{-1}: 3568 (O-H), 1647 (C=O), 1638 (C=N). ^{1}H-NMR (400 MHz, $CDCl_3$): δ 5.16 (s, 1H, O-H), 6.72 (d, 1H, J = 10.4 Hz, 3-H), 6.95 (dd, 4H, J = 8.8, 8.8 Hz, 2'-H + 3'-H + 5'-

H + 6'-H), 7.31 (d, 1H, *J* = 8.3 Hz, 6-H), 7.37 (d, 1H, *J* = 10.4 Hz, 2-H), 7.53 (dd, 1H, *J* = 7.8, 8.0 Hz, 7-H), 7.71 (d, 1H, *J* = 7.3 Hz, 8-H), 14.20 (s, 1H, OH). ^{13}C-NMR (100 MHz, $CDCl_3$): δ 114.59, 116.11(2C), 118.28, 123.65, 124.28 (2C), 130.22, 131.53, 132.86, 135.09, 138.77, 154.70, 159.30, 160.72, 185.18; HRMS (APCI) calcd. for $C_{16}H_{11}NO_3$: 266.08172 $[M+H]^+$; found 266.08029.

5-Hydroxy-4-((4-methoxyphenyl)imino)naphthalen-1(4H)-one (**7**): Prepared from **1** and 4-methoxyphenylamine, in 47 and 34% yield with solar light green LEDs respectively; brown solid, mp 147-149 °C; IR (KBr) $\nu_{máx}$ cm^{-1}: 3630 (O-H), 1640 (C=O), 1624 (C=N). ^{1}H-NMR (400 MHz, $CDCl_3$): δ 3.86 (s, 1H, OMe), 6.71 (d, 1H, *J* = 10.5 Hz, 3-H), 7.01 (dd, 4H, *J* = 9.1, 9.0 Hz, 2'-H + 3'-H + 5'-H + 6'-H), 7.30 (d, 1H, *J*= 8.2 Hz, 6-H), 7.37 (d, 1H, *J* = 10.5 Hz, 2-H), 7.53 (dd, 1H, *J* = 7.9, 7.9 Hz, 7-H), 7.70 (d, 1H, *J* = 7.6 Hz, 8-H), 14.21 (s, 1H, OH). ^{13}C-NMR (100 MHz, $CDCl_3$): δ 55.59, 114.56, 114.59, 116.02, 118.20, 123.56, 124.12 (2C), 130.20, 131.55, 132.78, 135.04, 138.69, 158.61, 159.20, 160.71, 185.10; HRMS (APCI) calcd. for $C_{17}H_{13}NO_3$: 280.09737 $[M+H]^+$; found 280.09625.

4-((2,5-Dimethoxyphenyl)imino)-5-hydroxynaphthalen-1(4H)-one (**8**): Prepared from **1** and 2,5-dimethoxyphenylamine, in 60 and 43% yield with solar light and green LEDs respectively; brown solid, mp 260-262 °C; IR (KBr) $\nu_{máx}$ cm^{-1}: 3285 (O-H), 1645 (C=O), 1635 (C=N). ^{1}H-NMR (400 MHz, $CDCl_3$): δ 3.79 (s, 3H, OMe), 3.86 (s, 3H, OMe), 6.65 (m, 1H, 4'-H or 3'-H), 6.71 (s, 1H, Hz, 6'-H), 6.79 (m, 1H, 3'-H or 4'-H), 6.83 (d, 1H, *J* = 8.9 Hz, 3-H), 6.93 (d, 1H, *J* = 8.9 Hz, 2-H), 7.34 (d, 1H, *J* = 8.3 Hz, 6-H), 7.51 (dd, 1H, *J* = 7.9, 8.0 Hz, 7-H), 7.78 (d, 1H, *J* = 7.6 Hz, 8-H), 15.34 (s, 1H, OH). ^{13}C-NMR (100 MHz, $CDCl_3$): δ 55.71, 56.24, 97.21, 105.07, 109.39, 111.05, 113.31, 118.56, 125.13, 128.60, 131.92, 136.17, 140.04, 144.67, 153.89, 161.58, 161.82, 185.10; HRMS (APCI) calcd. for $C_{18}H_{15}NO_4$: 310.10794 $[M+H]^+$; found 310.10712.

5-Hydroxy-4-((3,4,5-trimethoxyphenyl)imino)naphthalen-1(4H)-one (**9**): This compound was prepared from **1** and 3,4,5-trimethoxyphenylamine, in 59 and 38% yield with solar light and green LEDs respectively; red solid, mp 148- 150 °C; IR (KBr) $\nu_{máx}$ cm^{-1}: 3568 (O-H), 1689 (C=O), 1627 (C=N). ^{1}H-NMR (400

MHz, $CDCl_3$): δ 3.87 (s, 6H, OMe), 3.89 (s, 3H, OMe), 6.25 (s, 2H, 2'-H + 6'-H), 6.72 (d, 1H, *J* = 10.4 Hz, 3-H), 7.32 (d, 1H, *J* = 8.2 Hz, 6-H), 7.34 (d, 1H, *J* = 10.4 Hz, 2-H), 7.55 (dd, 1H, *J* = 7.9, 8.0 Hz, 7-H), 7.71 (d, 1H, *J* = 7.5 Hz, 8-H), 13.86 (s, 1H, OH). ^{13}C-NMR (100 MHz, $CDCl_3$): δ 56.28 (2C), 61.09, 99.62 (2C), 114.56, 115.69, 118.43, 123.71, 130.39, 131.57, 133.14, 135.42, 136.36, 141.71, 153.69, 160.13, 160.64, 184.99; HRMS (APCI): calcd. for $C_{19}H_{17}NO_5$: 340.11850 $[M+H]^+$; found 340.11746.

5-Hydroxy-4-((4-hydroxyphenyl)imino)naphthalen-1(4H)-one (**10**): Prepared from **5** and 4-hydroxyphenylamine, in 88 and 82% yield with solar light and green LEDs respectively; red solid, mp 258-259 ºC; IR (KBr) $\nu_{máx}$ cm^{-1}: 3244 (NH), 1691 (C=O), 1655 (C=O), 1643 (C=N). ^{1}H-NMR (400 MHz, DMSO-d_6): δ 2.15 (s, 3H, COMe), 6.75 (d, 1H, *J* = 10.5 Hz, 3-H), 6.92 (d, 2H, *J* = 8.6 Hz, 3'-H + 5'-H), 7.04 (d, 2H, *J* = 8.6 Hz, 2'-H + 6'-H), 7.42 (d, 1H, *J* = 10.5 Hz, 2-H), 7.66 (dd, 1H, *J* = 8.0, 8.0 Hz, 7-H), 7.77 (d, 1H, *J* = 7.4 Hz, 6-H), 8.94 (d, 1H, *J* = 8.2 Hz, 8-H), 9.88 (s, 1H, 4'-OH), 13.41 (s, 1H, 5-OH). ^{13}C-NMR (400 MHz, DMSO-d_6): δ 26.13, 116.37 (2C), 118.17, 121.01, 124.59, 124.77 (2C), 131.96, 132.01, 132.30, 132.80, 138.76, 139.91, 157.26, 169.42, 184.99, 185.03; HRMS (APCI) calcd. for $C_{18}H_{14}N_2O_3$: 306.10044 $[M+H]^+$; found 306.10189.

X-ray crystallography:

X-ray crystallographic data were collected at 293 K with Cu Kα (1.5418 Å) using an Oxford diffraction CCD area-detector diffractometer equipped with graphite monochromator. The Cris Alis PRO software [1] was used for data collection data reduction and cell refinement. The structure was solved by direct methods and refined by full-matrix least-squares calculations using SHELXL [2] software. All the non-H atoms were refined in the anisotropic approximation against F2 of all reflections. The H-atoms were placed at their calculated positions and refined isotropic approximation. The crystallographic parameters are given in Table S1.

The crystallographic information of the compound is deposited to Cambridge Crystallographic Database and has the CCDC number 1015027

References:

[1] Agilent (2010). CrysAlis PRO. Agilent Technologies, Yarnton, England.

[2] Sheldrick, G. M. (2008). Acta Cryst. A64, 112–122.

Table S1. Crystal data and structure refinement for compound 6.

Identification code	shelx
Empirical formula	$C_{16}H_{11}NO_3$
Formula weight	266.08
Temperature	293(2) K
Wavelength	1.5418 ≈
Crystal system	Triclinic
Space group	P -1
Unit cell dimensions	a = 7.445(3) ≈ α= 109.945(3)∞.<br>b = 13.785(4) ≈ β= 101.686(4)∞.<br>c = 14.088(6) ≈ γ = 101.735(3)∞.
Volume	1271.3(8) ≈3
Z	2
Density (calculated)	1.433 Mg/m^3
Absorption coefficient	0.844 mm^{-1}
F(000)	572
Theta range for data collection	3.493 to 61.581∞.
Index ranges	-6<=h<=8, -15<=k<=15, -16<=l<=14
Reflections collected	6849
Independent reflections	3883 [R(int) = 0.0122]
Completeness to theta = 67.680∞	84.7 %
Refinement method	Full-matrix least-squares on F^2
Data / restraints / parameters	3883 / 0 / 388
Goodness-of-fit on F^2	1.045
Final R indices [I>2sigma(I)]	R1 = 0.0337, wR2 = 0.0872
R indices (all data)	R1 = 0.0392, wR2 = 0.0928
Extinction coefficient	n/a
Largest diff. peak and hole	0.300 and -0.160 $e.≈^{-3}$

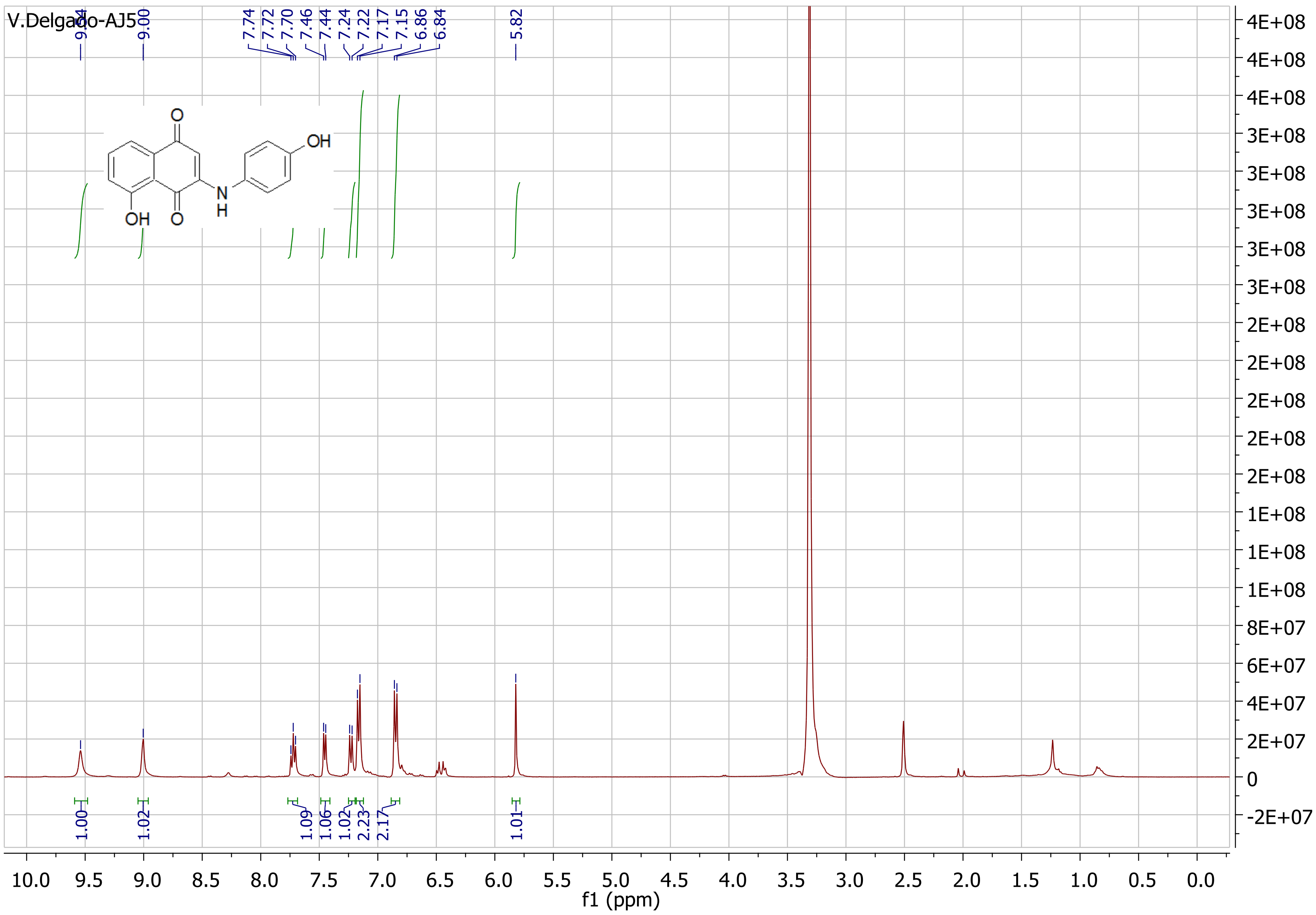
V.Delgado-AJ5
9.54
9.00
7.74
7.72
7.70
7.46
7.44
7.24
7.22
7.17
7.15
6.86
6.84
5.82
O
OH
N
H
OH
O
1.00
1.02
1.09
1.06
1.02
2.23
2.17
1.01
f1 (ppm)

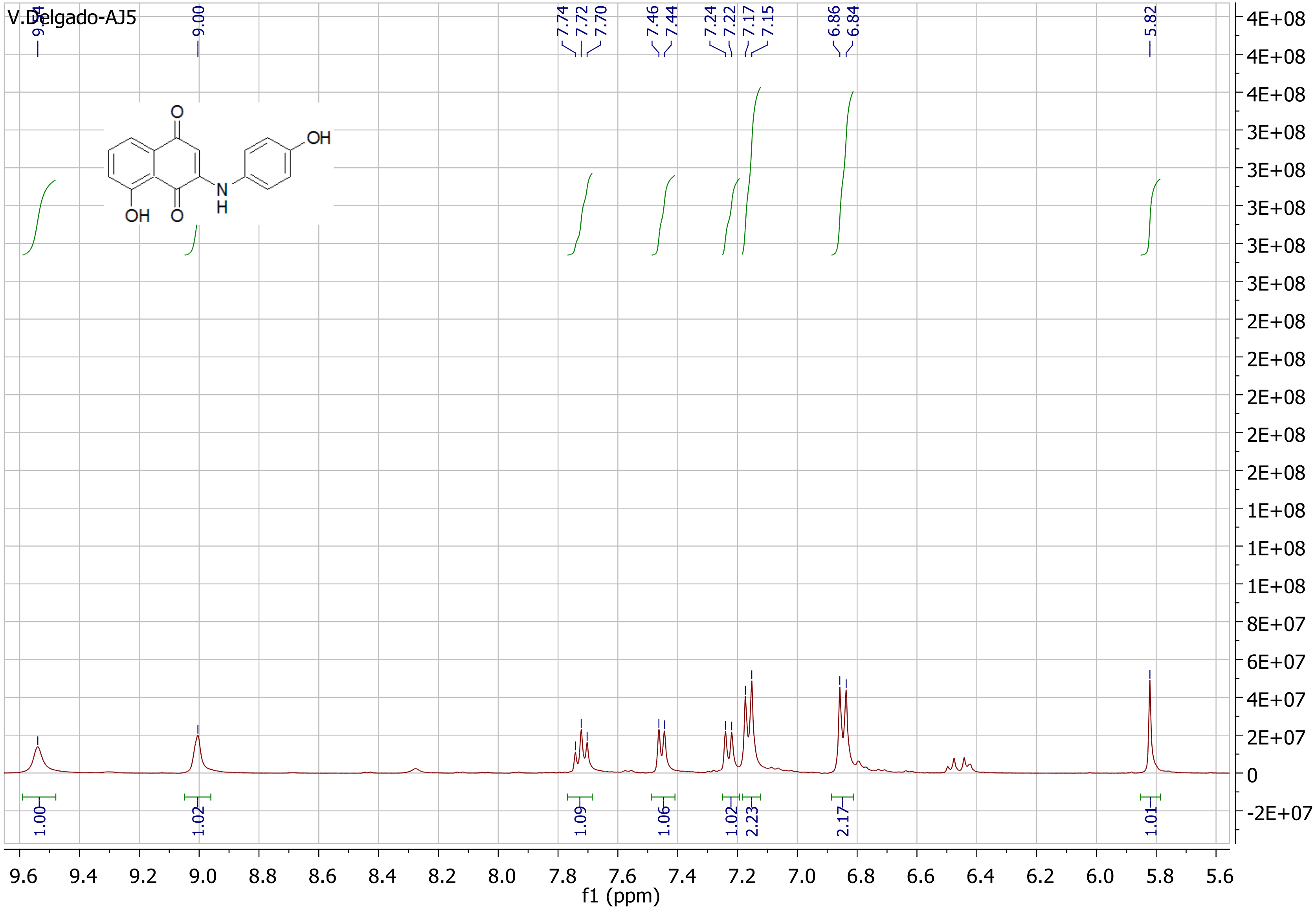
V.Delgado-AJ5
9.54
9.00
7.74
7.72
7.70
7.46
7.44
7.24
7.22
7.17
7.15
6.86
6.84
5.82
OH
N
H
OH
O
O
1.00
1.02
1.09
1.06
1.02
2.23
2.17
1.01
f1 (ppm)

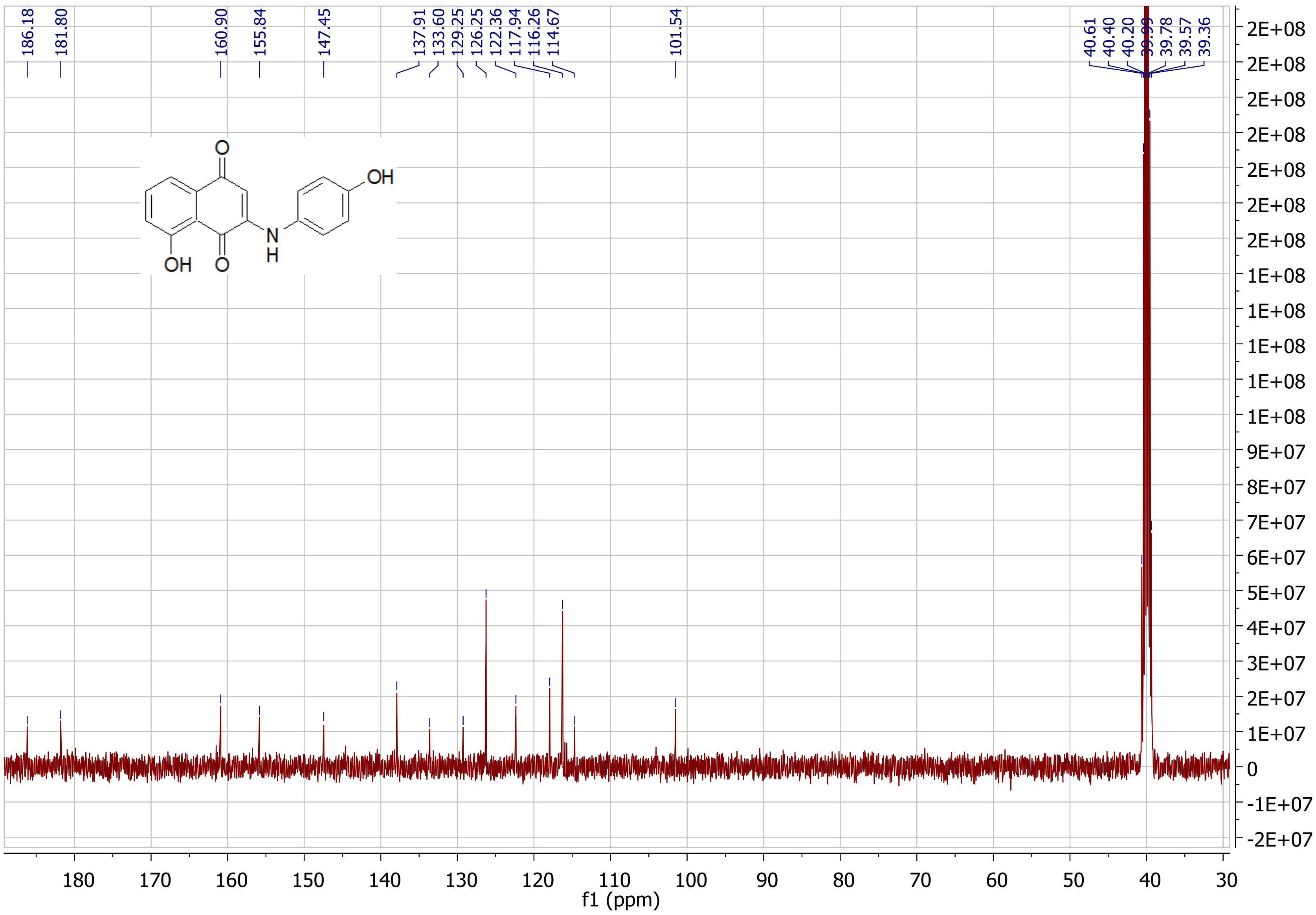
186.18
181.80
160.90
155.84
147.45
137.91
133.60
129.25
126.25
122.36
117.94
116.26
114.67
101.54
40.61
40.40
40.20
39.99
39.78
39.57
39.36
O
OH
N
H
OH
O
2E+08
1E+08
9E+07
8E+07
7E+07
6E+07
5E+07
4E+07
3E+07
2E+07
1E+07
0
-1E+07
-2E+07
180
170
160
150
140
130
120
110
100
90
80
70
60
50
40
30
f1 (ppm)

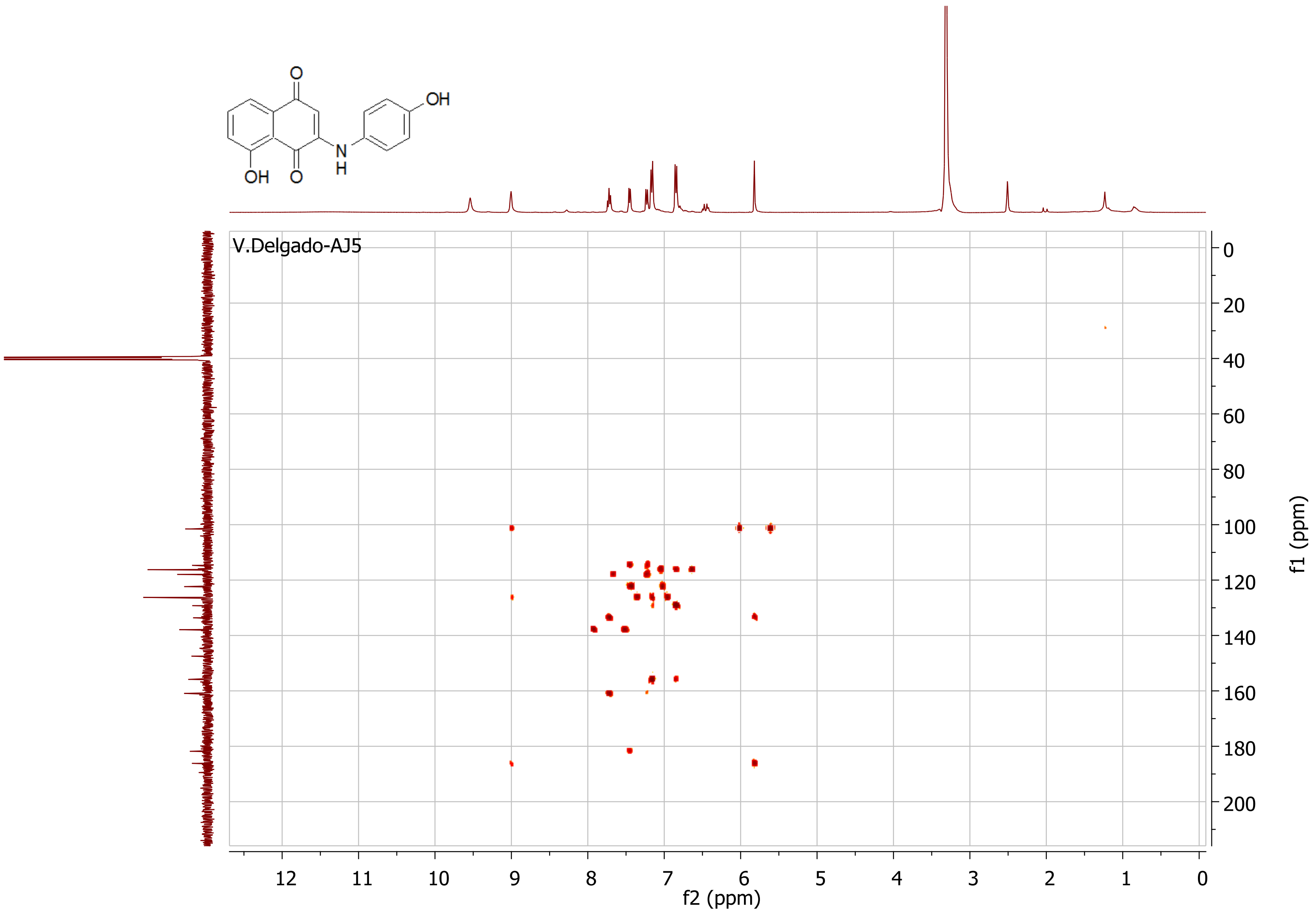
O
OH
N
H
OH
O
V.Delgado-AJ5
f2 (ppm)
f1 (ppm)

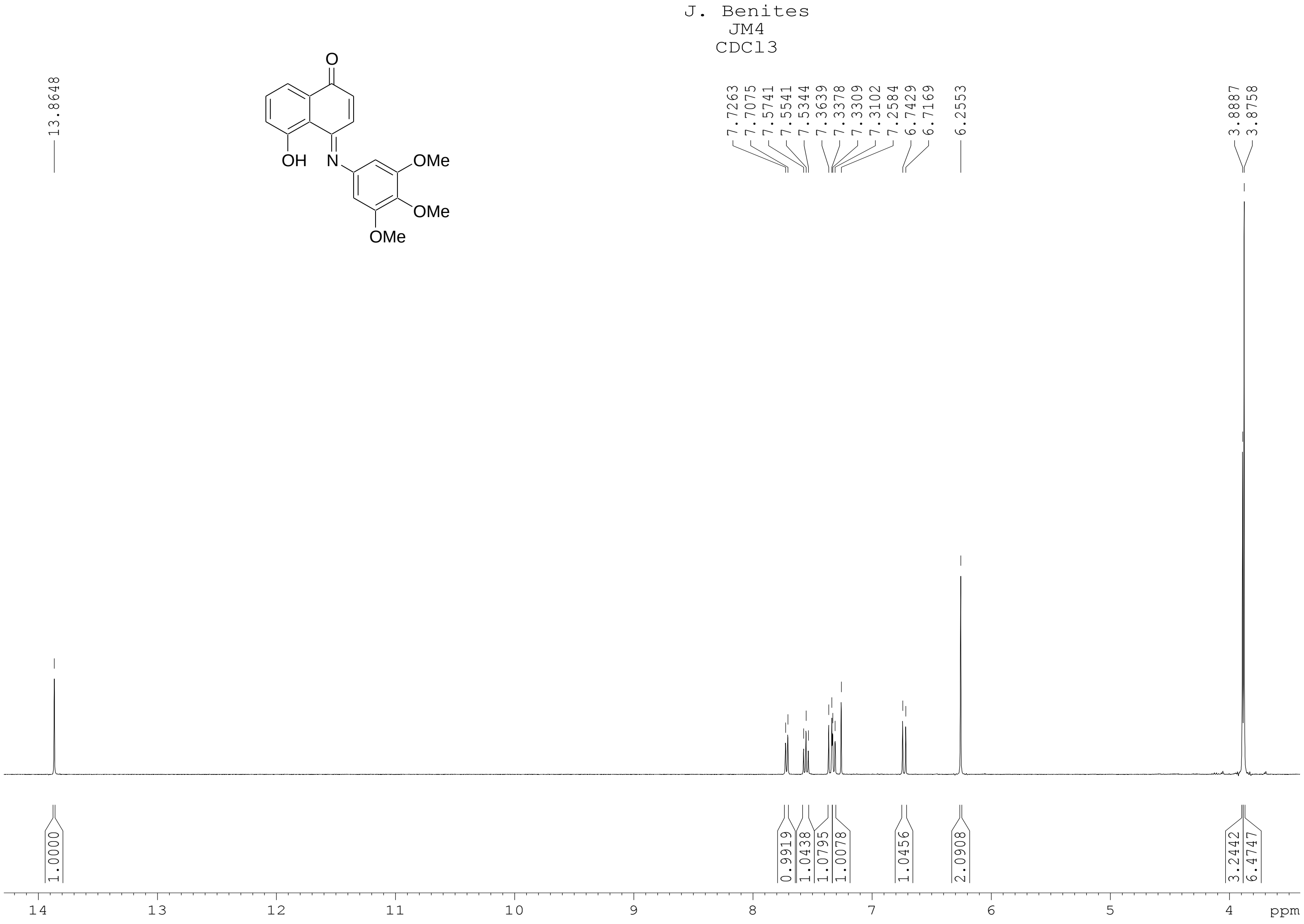

NAME	Julio Benites	
EXPNO	81	
PROCNO	1	
Date_	20120823	
Time	10.17	
INSTRUM	spect	
PROBHD	5 mm BBI 1H-BB	
PULPROG	zg30	
TD	32768	
SOLVENT	CDCl3	
NS	8	
DS	0	
SWH	8012.820	Hz
FIDRES	0.244532	Hz
AQ	2.0447731	sec
RG	322.5	
DW	62.400	usec
DE	6.00	usec
TE	294.2	K
D1	1.00000000	sec
TD0	1	

======== CHANNEL f1 ========

NUC1	1H	
P1	6.50	usec
PL1	0.00	dB
SFO1	400.1338012	MHz
SI	16384	
SF	400.1300099	MHz
WDW	no	
SSB	0	
LB	0.00	Hz
GB	0	
PC	1.00	

J. Benites
JM4
CDCl3

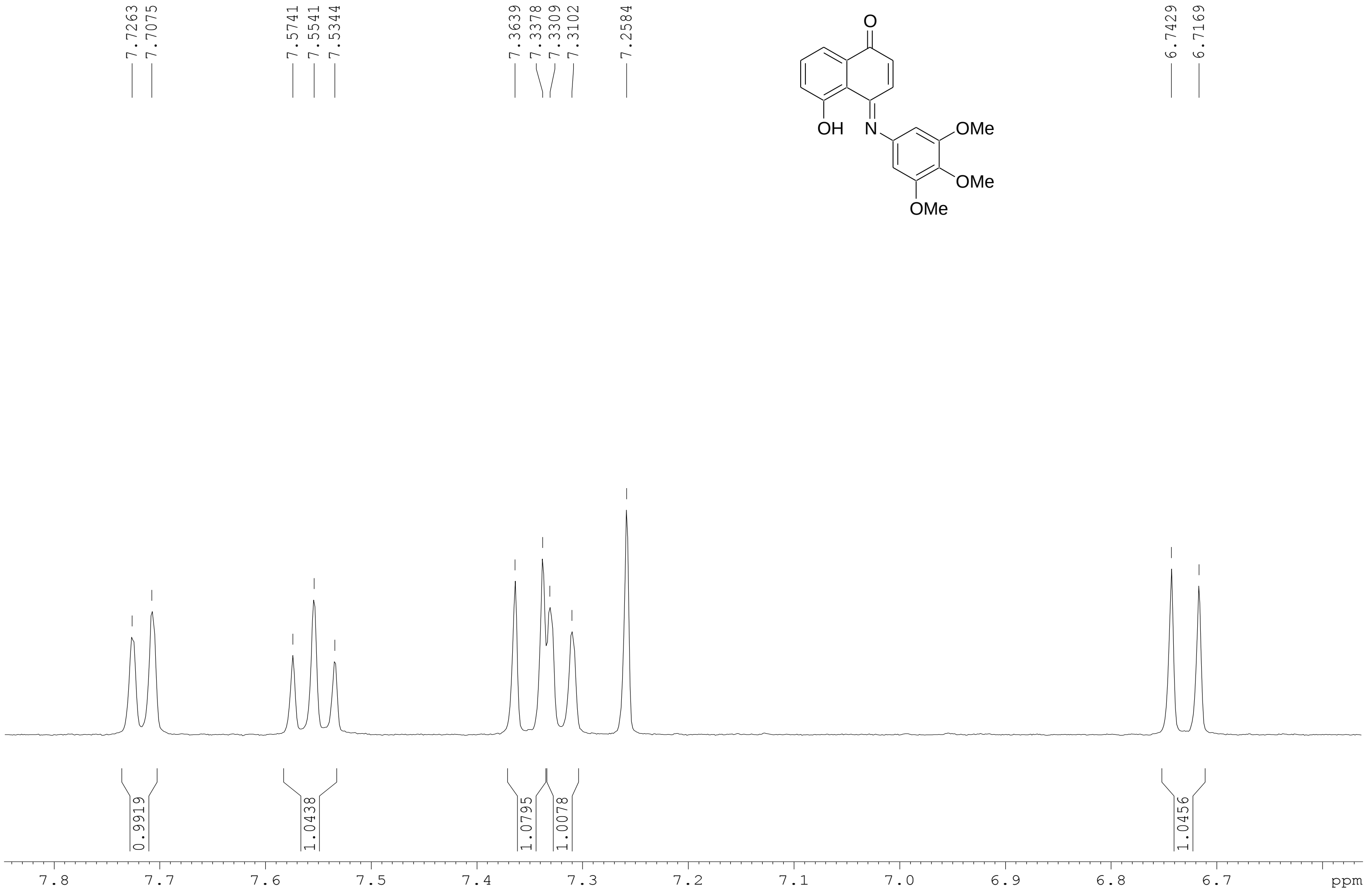

NAME	Julio Benites	
EXPNO	81	
PROCNO	1	
Date_	20120823	
Time	10.17	
INSTRUM	spect	
PROBHD	5 mm BBI 1H-BB	
PULPROG	zg30	
TD	32768	
SOLVENT	CDCl3	
NS	8	
DS	0	
SWH	8012.820	Hz
FIDRES	0.244532	Hz
AQ	2.0447731	sec
RG	322.5	
DW	62.400	usec
DE	6.00	usec
TE	294.2	K
D1	1.00000000	sec
TD0	1	

======== CHANNEL f1 ========

NUC1	1H	
P1	6.50	usec
PL1	0.00	dB
SFO1	400.1338012	MHz
SI	16384	
SF	400.1300099	MHz
WDW	no	
SSB	0	
LB	0.00	Hz
GB	0	
PC	1.00	

J. Benites
JM4
CDCl3

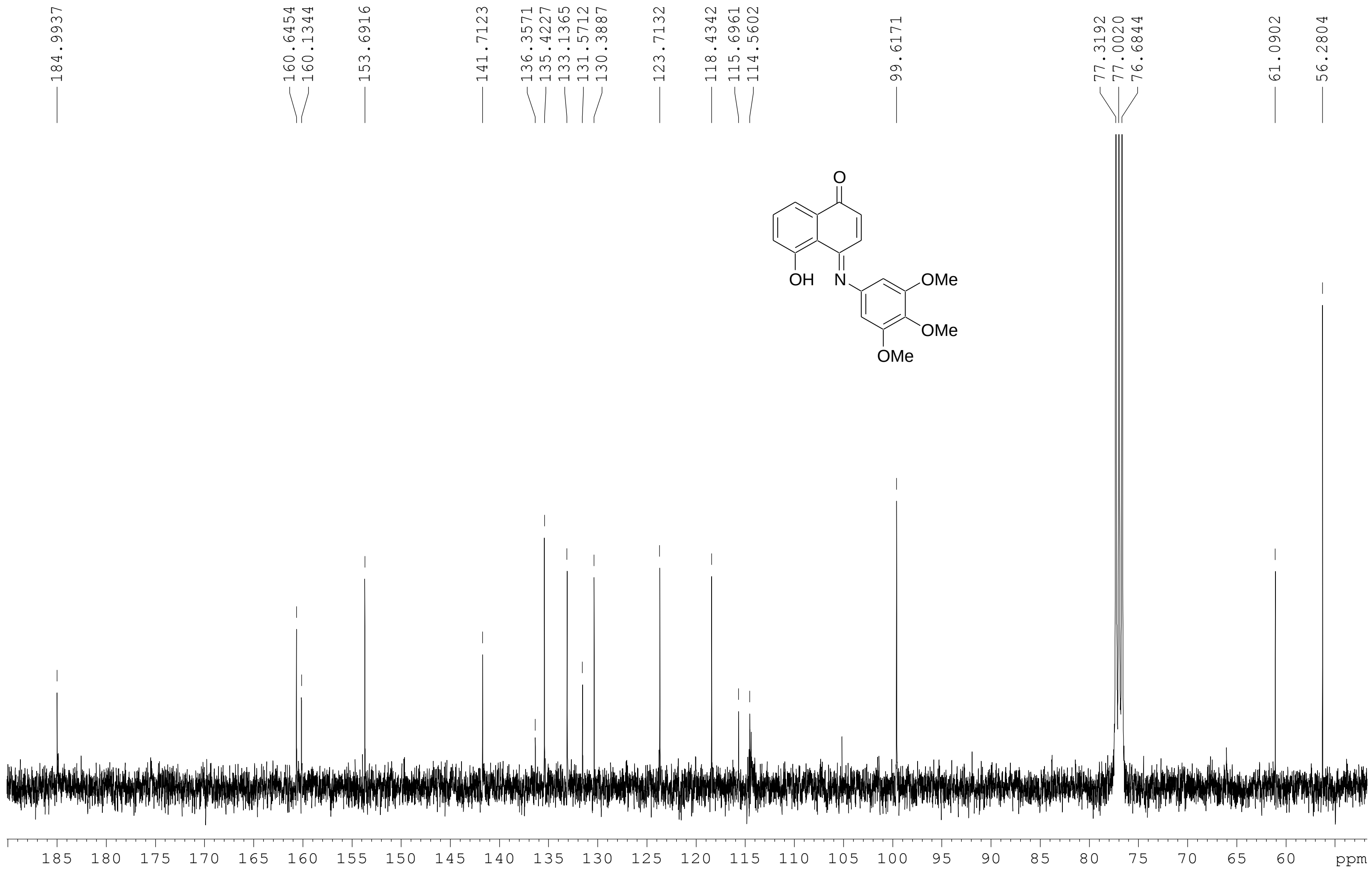

NAME	Julio Benites
EXPNO	96
PROCNO	1
Date_	20120828
Time	11.51
INSTRUM	spect
PROBHD	5 mm BBI 1H-BB
PULPROG	zgpg30
TD	32768
SOLVENT	CDCl3
NS	3079
DS	0
SWH	23980.814 Hz
FIDRES	0.731836 Hz
AQ	0.6832628 sec
RG	35.9
DW	20.850 usec
DE	6.00 usec
TE	292.2 K
D1	2.00000000 sec
D11	0.03000000 sec
TD0	1
======== CHANNEL f1 ========	
NUC1	13C
P1	14.00 usec
PL1	-6.00 dB
SFO1	100.6242995 MHz
======== CHANNEL f2 ========	
CPDPRG2	waltz16
NUC2	1H
PCPD2	85.00 usec
PL2	6.00 dB
PL12	23.00 dB
PL13	23.00 dB
SFO2	400.1316005 MHz
SI	32768
SF	100.6127714 MHz
WDW	EM
SSB	0
LB	1.00 Hz
GB	0
PC	1.40

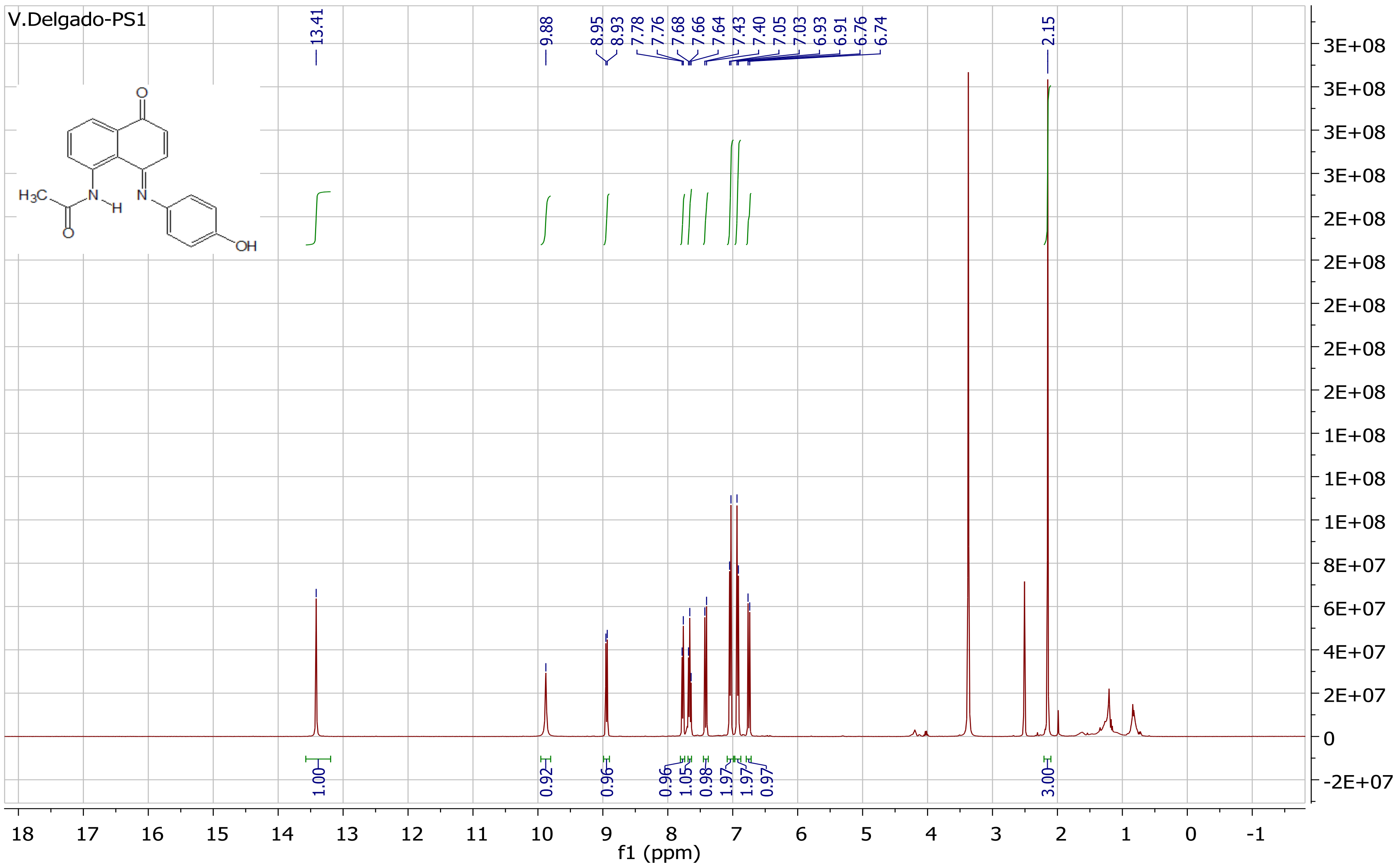
V.Delgado-PS1
13.41
9.88
8.95
8.93
7.78
7.76
7.68
7.66
7.64
7.43
7.40
7.05
7.03
6.93
6.91
6.76
6.74
2.15
1.00
0.92
0.96
0.96
1.05
0.98
1.97
1.97
0.97
3.00
f1 (ppm)

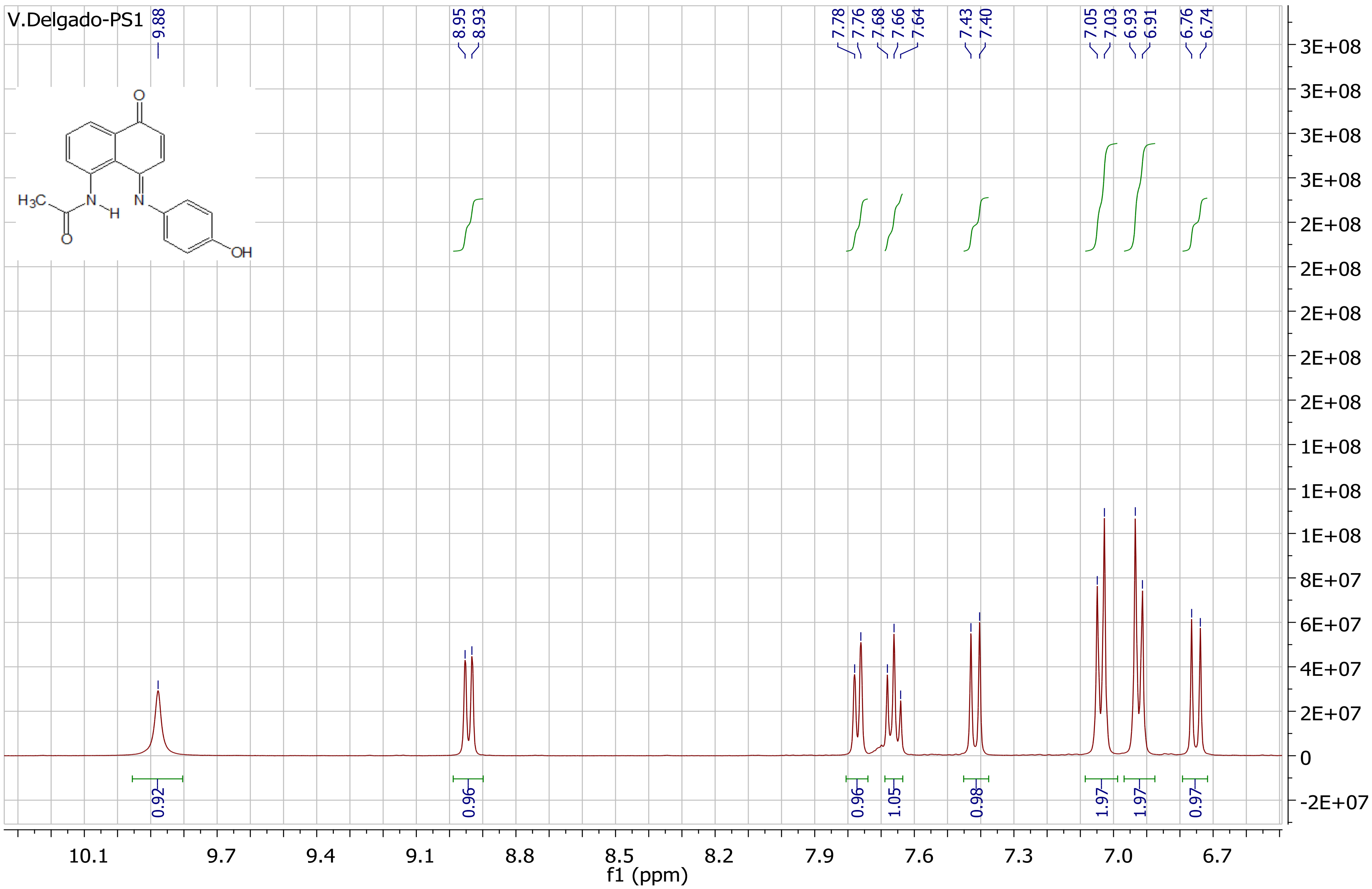

V.Delgado-PS1
9.88
8.95
8.93
7.78
7.76
7.68
7.66
7.64
7.43
7.40
7.05
7.03
6.93
6.91
6.76
6.74
0.92
0.96
0.96
1.05
0.98
1.97
1.97
0.97
f1 (ppm)

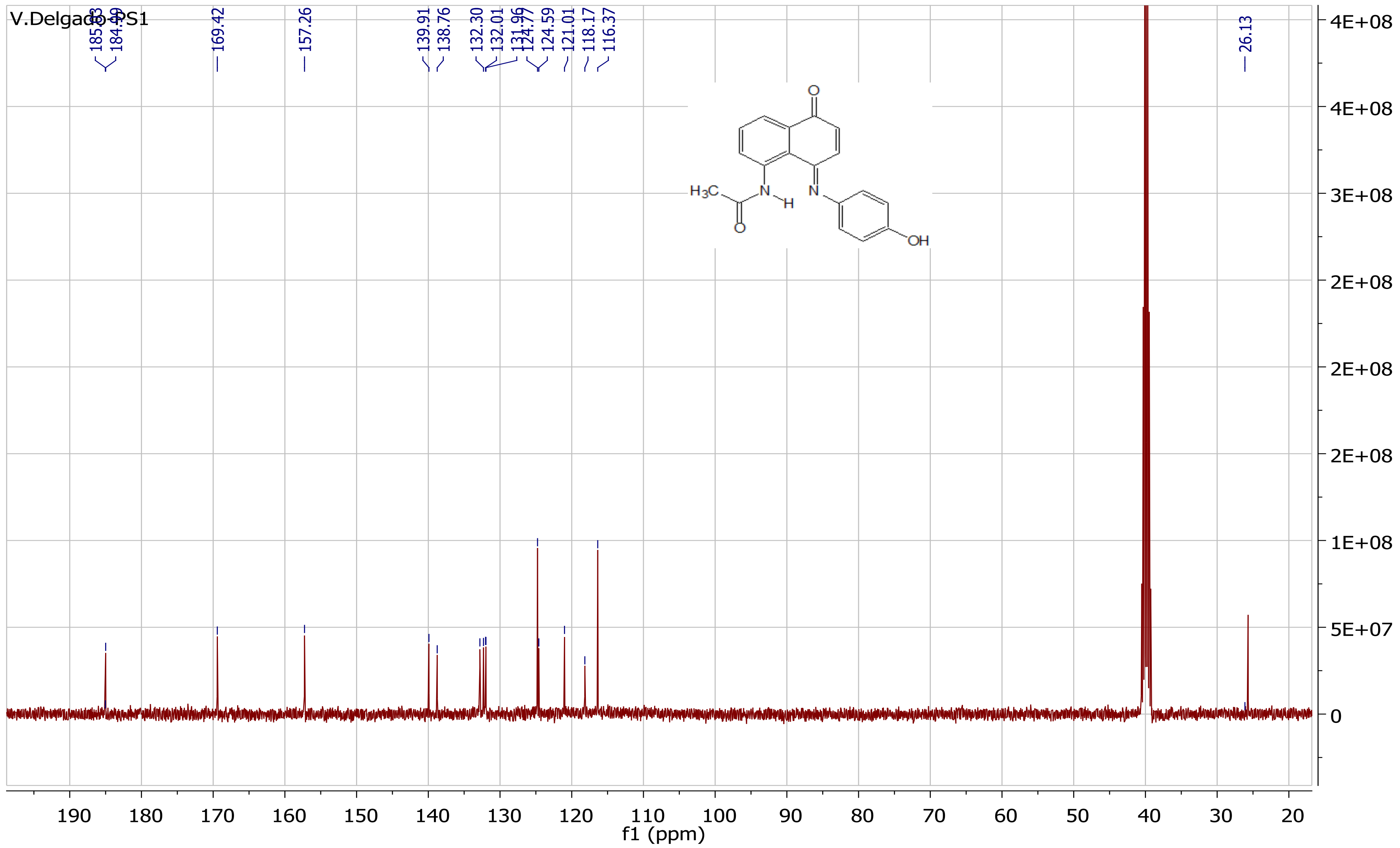
V.Delgado-PS1
169.42
157.26
139.91
138.76
132.30
132.01
131.96
124.77
124.59
121.01
118.17
116.37
26.13
4E+08
4E+08
3E+08
2E+08
2E+08
2E+08
1E+08
5E+07
0
190
180
170
160
150
140
130
120
110
100
90
80
70
60
50
40
30
20
f1 (ppm)
O
H_3C
N
H
N
O
OH
